# Recovering predators link aquatic and terrestrial ecosystems: River otters subsidize coyotes with carrion

**DOI:** 10.1002/ece3.11444

**Published:** 2024-06-17

**Authors:** Francis D. Gerraty, Terence Carroll, Skyler Williams, Megan Isadore

**Affiliations:** ^1^ Department of Ecology and Evolutionary Biology University of California—Santa Cruz Santa Cruz California USA; ^2^ River Otter Ecology Project Forest Knolls California USA

**Keywords:** carrion, ecological connectivity, kleptoparasitism, predator effects, scavenging

## Abstract

Despite global declines in the abundance and distribution of predators, conservation and reintroduction efforts are increasingly leading to predator recoveries. Unexpected species interactions and ecological consequences often arise when these predator recoveries occur. Here, we describe a novel species interaction in which coyotes (*Canis latrans*) kleptoparasitize North American river otters (*Lontra canadensis*) in a region of river otter recovery along the north‐central California coast, USA. We describe eight observations of coyotes scavenging otter‐killed waterbird carrion, including one observation in which river otters aggressively defended their prey from a coyote kleptoparasite. These observations highlight the importance of carrion provisioning as an overlooked pathway through which river otters facilitate nutrient subsidies to terrestrial scavengers. This behavior may have ecological implications including effects on the abundance, behavior, and health of scavengers as well as their interspecific interactions. We propose hypotheses and questions regarding these ecological consequences to guide further investigations into the cross‐ecosystem impacts of recovering river otter populations.

## Graphical abstract

We describe a novel species interaction in which coyotes (*Canis latrans*) kleptoparasitize North American river otters (*Lontra canadensis*) in a region of river otter recovery along the north‐central California coast, USA. We describe eight observations of coyotes scavenging otter‐killed waterbird carrion, including one observation in which river otters aggressively defended their prey from a coyote kleptoparasite. These observations highlight the importance of carrion provisioning as an overlooked pathway through which river otters facilitate nutrient subsidies to terrestrial scavengers, and we outline hypotheses and questions regarding the ecological consequences of this cross‐ecosystem linkage.

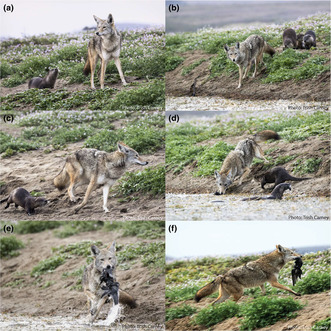

## 1 INTRODUCTION

Predator populations have declined considerably across previous centuries due to habitat destruction, direct exploitation, and other anthropogenic impacts (Ripple et al., 2014, Young et al., 2016). However, exceptions to this trend are increasingly numerous, as conservation and reintroduction efforts facilitate predator population recovery and habitat recolonization (Ingeman et al., 2022). Unexpected species interactions and ecological consequences are often revealed through these recoveries, such as predator modulation of diverse ecosystem processes including primary production, disease dynamics, wildfire, biogeochemical cycling, and carbon sequestration (Estes et al., 2011; Levi et al., 2012; Roffler et al., 2023; Schmitz et al., 2010; Wilmers et al., 2003, 2012). Predator recoveries therefore provide unique natural experiments to examine how ecological processes and ecosystem functions compare among intact and defaunated landscapes (Ingeman et al., 2022; Young et al., 2016).

The historic range of North American river otters (*Lontra canadensis*; hereafter “river otters”) once spanned much of the North American continent—from what is now the southern United States to the arctic (Bricker et al., 2022; Hall, 1981). Following European colonization, overexploitation and habitat destruction drove pronounced population declines and extirpations across much of this range. Despite these declines, river otter populations have grown substantially in the past few decades and have exhibited widespread range expansion due to regulations on harvest, habitat restoration, and reintroduction initiatives (Bricker et al., 2022; Roberts et al., 2020). In coastal north‐central California, USA, river otters were extirpated from much of their historic range prior to the 1930s (Grinnell et al., 1937), but began to recover in the 1970s (Schempf & White, 1977). The species is now present in freshwater, marine, and terrestrial habitats (Bouley et al., 2015), where it preys upon fish, birds, amphibians, and invertebrates (Cosby & Szykman Gunther, 2021; Penland & Black, 2009).

As boundary‐spanning predators, river otters act as important vectors of cross‐ecosystem nutrient subsidies. River otter‐mediated nutrient flows often occur through scent marking (i.e., defecation and urination) in terrestrial habitat patches known as “latrines” (Ben‐David et al., 2005). Marine and freshwater‐derived nutrients aggregate in these latrines, leading to enhanced soil and vegetation nutrient levels and plant growth (Ben‐David et al., 1998; Crait & Ben‐David, 2007). In addition to scent marking, river otters can contribute aquatic nutrients to terrestrial ecosystems by provisioning scavengers with otter‐predated carcasses. However, while several species including bobcats (*Lynx rufus*) and bald eagles (*Haliaeetus leucocephalus*) have been documented stealing and/or scavenging carcasses killed by river otters, the extent and magnitude of this subsidy pathway remains unknown (Bergan, 1990; Taylor, 1992).

Here, we describe seven instances of coyotes (*Canis latrans*) scavenging waterbird carcasses provisioned by river otters in north‐central California between 2016 and 2023. One of these observations includes direct evidence of competitive aggression between river otters and coyotes prior to and during the scavenging event, suggesting that coyotes kleptoparasitize otters in addition to scavenging otter‐cached carrion. We also describe an additional observation in which river otters successfully defended their prey from a coyote kleptoparasite. We propose that carrion provisioning by river otters may be an overlooked pathway of aquatic‐to‐terrestrial nutrient flow throughout the species' range, and lay out questions and hypotheses about the resulting ecological consequences. Taken together, our observations provide an opportunity to consider the pathways through which recovering, boundary‐spanning predators serve as catalysts of ecosystem connectivity.

## 2 METHODS

### 2.1 Study area

**FIGURE 1:**
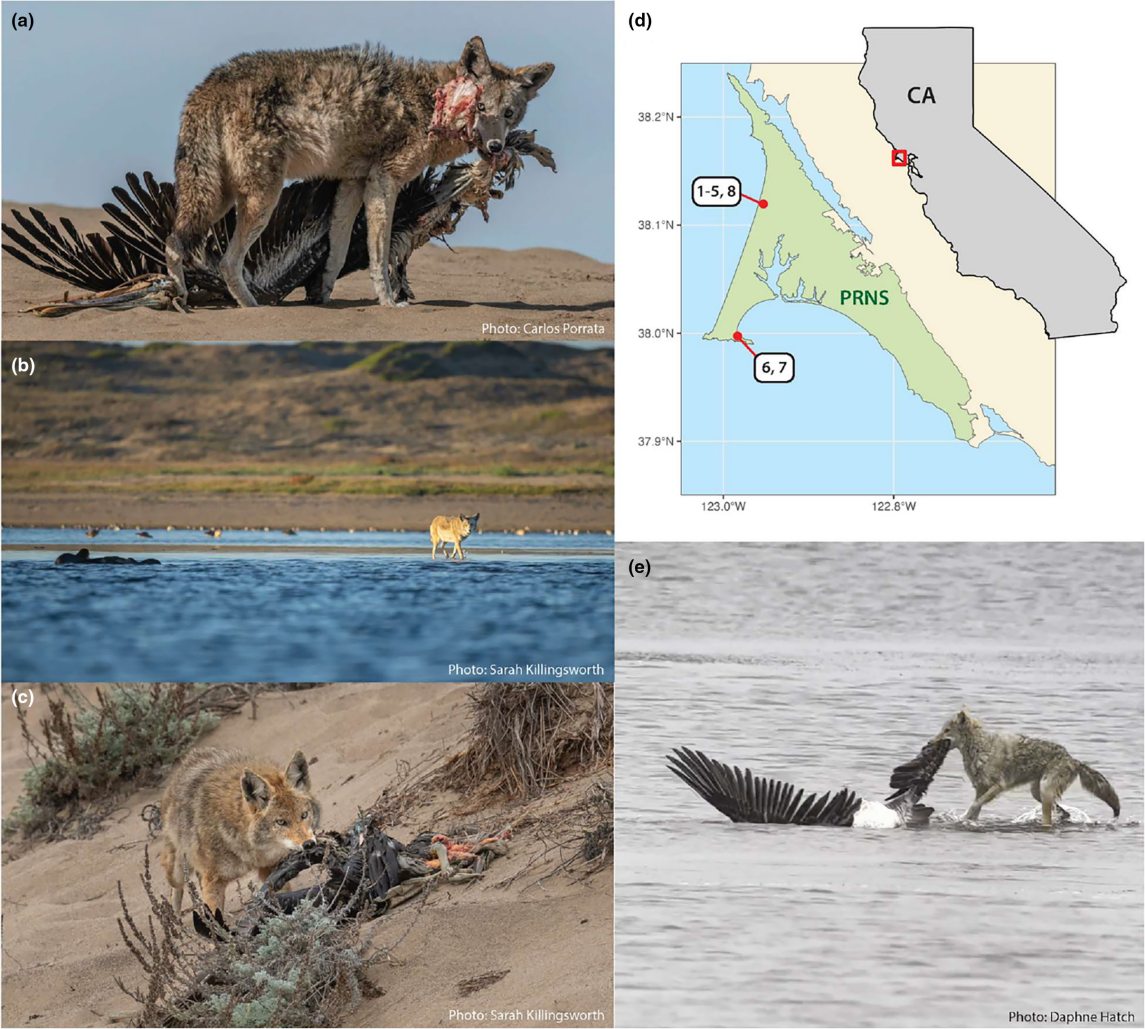
(a) Coyote scavenging brown pelican carcass at Abbotts Lagoon on 17 December 2021 (Observation #3). Photograph: Carlos Porrata. (b) Coyote wading into Abbotts Lagoon toward river otters feeding on a brown pelican on 23 November 2023 (Observation #8). Photograph: Sarah Killingsworth. (c) Coyote scavenging otter‐predated brown pelican carcass at Abbotts Lagoon on 6 October 2021 (Observation #2). Photograph: Sarah Killingsworth. (d) Map of the study area, Point Reyes National Seashore, with red points representing locations of observations. Number labels correspond to observation numbers in Table 1. (e) Coyote wading into Abbotts Lagoon to retrieve otter‐predated brown pelican on 9 October 2022 (Observation #5). Photograph: Daphne Hatch.

Our study area, Point Reyes National Seashore (PRNS), is located along the north‐central coast of California and encompasses a diverse coastal landscape featuring sandy beaches, lagoons, estuarine wetlands, rocky shorelines, dunes, grasslands, coastal scrub, and forests (Figure 1d). Following historical extirpation, natural range expansion by river otters has occurred across the San Francisco Bay Area, with the earliest documented observations of river otters returning to the PRNS area occurring around 1990 (Bouley et al., 2015). River otters in the region occupy a wide variety of fresh‐ and saltwater aquatic habitats including lakes and ponds, rivers, marshes, and the open ocean.

### 2.2 Community science observations and camera trap surveys

Since February 2012, we have solicited public observations of river otter sightings through an “otter spotter” community‐science platform (Bouley et al., 2015; see https://riverotterecology.org/otter‐spotter‐community‐based‐science/). The structured data collected through the platform includes the date and time of the observation, location, number of otters observed, other relevant data, and photos. A notes section allows the observer to report other information, which may include interspecific interactions. In tandem, since 2012 we have monitored six active river otter latrine sites or travel corridors in PRNS using motion‐triggered wildlife cameras (Bushnell HD Trophy Cam, Bushnell Products, Overland Park, KS; various models Browning Trail Cameras, Birmingham, AL) (Bouley et al., 2015; Carroll et al., 2020). At each site, we deployed 1–3 cameras that were programmed to record videos 30–60 s long and were serviced at least every 3 weeks. Lastly, we gathered observations of coyote‐otter interactions from local wildlife photographers and naturalists through word‐of‐mouth networking. While we identified coyotes as a focal scavenger for this investigation, it is likely that many other species exploit otter‐provisioned carrion in our study region and elsewhere.

## 3 RESULTS

In total, we documented seven independent instances of coyotes scavenging otter‐provisioned carcasses in Point Reyes National Seashore and one unsuccessful kleptoparasitism attempt (Table 1). Six of these observations were recorded at Abbotts Lagoon and contributed to the “otter spotter” community science platform or via word of mouth, and two of these observations were recorded by wildlife cameras at a latrine site in southwest Point Reyes (Figure 1d, Video 1). One coyote with a distinctive ear marking was observed scavenging otter‐provisioned carrion on at least two occasions, suggesting that this individual may exploit river otter prey remains with some regularity (Figure 1a,c; Table 1, Observations 2–3). The most documented river otter prey species scavenged by coyotes was the brown pelican (*Pelecanus occidentalis*; 5 independent observations), a large‐bodied and migratory seabird that is abundant in the region from July through December.

**TABLE 1:** Summary of eight documented instances of coyotes scavenging river otter‐provisioned carrion or attempting to kleptoparasitize river otters.

Observation number	Date & time	Location	Prey item	Description
1^a^	15 October 2016; 7:00	Abbotts Lagoon	American coot (*Fulica americana*)	At 7:00 on October 15, 2016, a river otter was observed returning to a group of five other otters at the Lagoon bank carrying a dead American coot in its mouth. Soon after, a coyote approached the same area, which appeared to be a food caching site, and retrieved a coot carcass from the waterline. The coyote returned to the location at least three times, each time grabbing additional waterbird carcasses. The otters were observed circling the coyote, charging aggressively, and attempting to nip at the coyote's feet—suggesting coyotes and otters compete over the otters' prey remains
2	6 October 2021	Abbotts Lagoon	Brown pelican (*Pelecanus occidentalis*)	On October 6, 2021, a coyote was observed removing a brown pelican carcass—which appeared to be a recent river otter kill—from Abbotts Lagoon and carrying the carcass into adjacent dunes, where it was scavenged
3	17 December 2021; 12:27	Abbotts Lagoon	Brown pelican (*Pelecanus occidentalis*)	At 12:27 on December 17, 2021, a coyote was observed removing a brown pelican carcass—which appeared to be a recent river otter kill— from Abbotts Lagoon and carrying the carcass into adjacent dunes, where it was scavenged
4	31 July 2022	Abbotts Lagoon	Brown pelican (*Pelecanus occidentalis*)	On July 31, 2022, a coyote was observed wading into Abbotts Lagoon to retrieve a brown pelican carcass that appeared to be a recent river otter kill. The coyote carried the carcass back to the shoreline, where it was scavenged
5	9 October 2022; 16:15	Abbotts Lagoon	Brown pelican (*Pelecanus occidentalis*)	At 16:00 on October 9, 2022, one river otter within a larger group was observed drowning a pelican and feeding on it approximately 140 m offshore in Abbotts Lagoon. At around 16:15, a coyote swam out to the location of the group of otters and pelican carcass. Prior to the coyote arriving at the pelican, the otters dispersed. The coyote proceeded to grab the pelican carcass and swim and walk the carcass to shore where it was scavenged
6	16 June 2023; 8:13	Elephant Seal Overlook	Unidentified waterbird	At 18:07 on June 9, 2023, we observed a river otter carrying a bird to a cache site and feeding on it with a motion‐triggered camera. One week later, at 8:13 on June 16, 2023, we documented a coyote visiting the cache site and scavenging a heavily consumed bird skeleton with approximately the same size and appearance
7	20 August 2023; 14:35	Elephant Seal Overlook	Unidentified waterbird	At 12:56 on August 20, 2023, we observed two otters feeding on a waterbird at a cache site with a motion‐triggered camera. At 14:35 the same day, we observed a coyote carrying a waterbird carcass with similar size and appearance away from the site
8^b^	23 November 2023	Abbotts Lagoon	Brown pelican (*Pelecanus occidentalis*)	On November 23, 2023, a coyote was observed wading into Abbotts Lagoon toward a group of river otters feeding on a pelican, just minutes after the otters killed it. The river otters chased off the coyote, which circled back once but was not observed accessing the carcass

^a^ Indicates confirmed kleptoparasitism.

^b^ Indicates attempted kleptoparasitism.

**VIDEO 1:** Video recordings of coyotes scavenging river otter prey remains corresponding to observations 5, 6, and 7 in Table 1. A high‐quality video can be accessed at https://doi.org/10.5281/zenodo.11391899.

**FIGURE 2:**
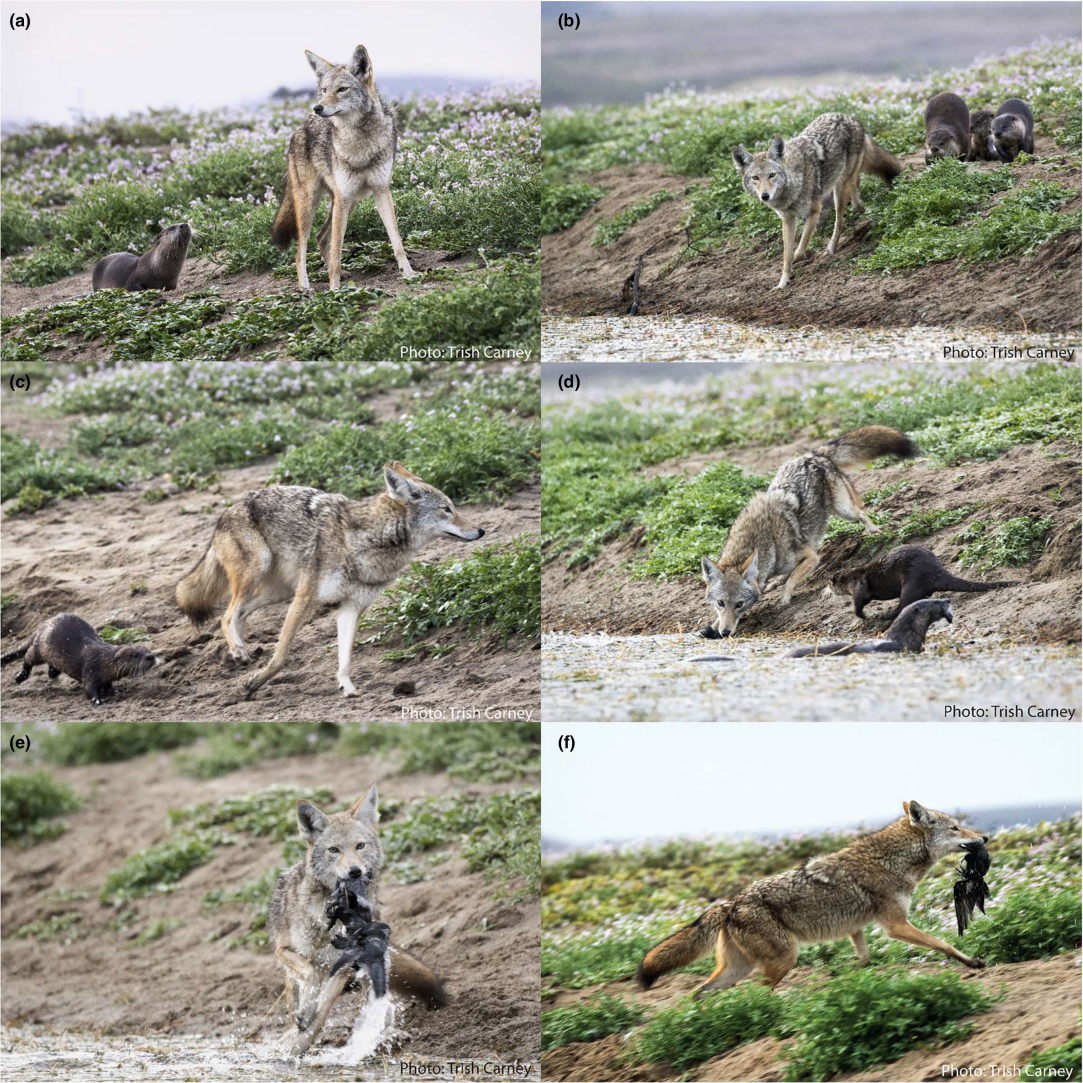
Coyote kleptoparasitizing river otters by stealing waterbird prey remains at Abbotts Lagoon on 15 October 2016 (Observation #1). River otters were observed displaying agonistic behavior including charging at the coyote (a, b) and nipping at its feet (c) in an attempt to prevent the coyote from accessing (d, e) and scavenging (f) the otter‐provisioned carrion. All photographs: Trish Carney.

One contributed observation includes direct evidence of aggressive behavior between river otters and coyotes, suggesting that coyotes directly kleptoparasitize river otters in addition to exploiting otter‐cached carrion. This kleptoparasitism event occurred in October 2016, when a coyote was seen approaching a group of six otters on the shore of Abbotts Lagoon and grabbing an otter‐predated American coot (*Fulica americana*) carcass from the waterline (Table 1, Observation 1; Figure 2). Several otters charged at the coyote and attempted to nip at its paws in a display of competitive aggression, but did not make physical contact. After carrying the bird carcass away to feed, the coyote returned to the river otter prey cache location two more times to retrieve additional waterbird carcasses. While only this observation included direct evidence of aggression between otters and coyotes, coyotes may also serve as kleptoparasites by displacing otters from their kills or removing cached prey remains (e.g., Table 1, Observation 5). However, kleptoparasitism attempts by coyotes may not always be successful. In one contributed observation, river otters chased an approaching coyote away from a fresh pelican kill and prevented the coyote from accessing the carcass (Table 1, Observation 8). Taken together, these observations suggest that interactions between river otters and coyotes may range from facilitative (i.e., otters provisioning discarded prey remains to coyotes) to competitive (i.e., otters and coyotes competing for otter‐killed prey).

## 4 DISCUSSION

Our results provide insight into the ecological consequences of habitat recolonization by North American river otters. The observations presented herein show that river otters, as boundary‐spanning predators, can drive cross‐ecosystem nutrient subsidies by provisioning prey carcasses derived from aquatic ecosystems to terrestrial scavengers. Although river otters are opportunistic predators, their prey selection is heavily influenced by the energetic costs of foraging in aquatic environments (Kruuk, 2006). As a result, they tend to favor larger, slower prey species and to employ repetitive use of successful foraging routes (Blundell et al., 2001; Day et al., 2015; Kruuk, 2006; Thompson & Stelle, 2014). In addition, river otters can gain a thermoregulatory advantage from catching larger prey that can be consumed on the shoreline (Kruuk, 2006). By transporting carrion onto shorelines, river otters provide terrestrial scavengers with access to prey items that would otherwise be largely inaccessible, and thus potentially increase the total availability of carrion biomass. While the magnitude and ecological influence of river otter provisioned carrion remains unquantified in coastal food webs, it is plausible that river otters strongly influence the quantity and distribution of decomposing carcasses in the ecosystems they inhabit.

We hypothesize that this is the case in our study region, where large‐bodied waterbirds—particularly brown pelicans—appear to be an important food source for river otters and consequently serve as carrion subsidies to terrestrial kleptoparasites. Waterbird predation by river otters is common (i.e., >50% frequency of occurrence of birds in otter scats) in several locations with seasonal waterbird aggregations in coastal California (Cosby & Szykman Gunther, 2021) and Alaska (Quinlan, 1983), but brown pelicans appear to be a novel prey species. This predator–prey relationship has only been documented in Del Norte County, CA (D Jaques, personal communication, November 30, 2023), at Rodeo Lagoon in Marin County, CA (Salman, 2007), and in PRNS as described herein. While the dynamics and consequences of this predator–prey interaction remain unquantified, we suspect that river otter predation is the primary cause of pelican mortality at Abbotts Lagoon and has consequently increased pelican carcass availability along the lagoon shoreline, where dozens of pelican carcasses are often observed throughout Fall–Winter. However, while brown pelicans may be particularly important to terrestrial kleptoparasites due to their large body size, other large‐bodied river otter prey such as fishes can serve a similar functional role in regions where waterbird predation is less common (Taylor, 1992).

Carrion provisioning by river otters directly enhances scavenger food resources, which may lead to shifts in scavenger space use, behavior, health, abundance, and interspecific interactions. For example, coyotes with predictable food subsidies often exhibit smaller home range size and greater abundance, which can decouple their abundance from local prey availability and drive subsequent shifts in food web dynamics (Hidalgo‐Mihart et al., 2004; Parsons et al., 2022; Rose & Polis, 1998). In addition, carrion subsidies may facilitate elevated conspecific tolerance by coyotes and drive associated shifts in social structure, or alternatively lead to increased agonistic behavior due to competition for carrion access (Newsome et al., 2015). Because river otter predation facilitates coyote consumption of waterbirds that are otherwise inaccessible, carrion‐subsidized coyotes may also be exposed to new sources of contaminants associated with aquatic ecosystems that can achieve high concentrations in avian predators (Elliott & Elliott, 2013).

In addition to increasing scavenger food resources, carcasses transferred to terrestrial ecosystems by river otters may increase local nutrient availability and generate biogeochemical hotspots, particularly in low‐productivity coastal dune habitats such as those surrounding Abbotts Lagoon (Johnson‐Bice et al., 2023). These carcass‐associated hotspots can generate novel interactions among local soil, plant, and invertebrate communities, potentially leading to landscape‐level consequences such as altered spatial heterogeneity (Monk & Schmitz, 2022).

However, these potential ecological effects would undoubtedly be modulated by spatial and temporal variation in the abundance and predictability of otter‐provisioned prey carcasses. In our case, interactions between otters, pelicans, and coyotes are almost certainly influenced by the patchy distribution and strong seasonality of brown pelican residence in the region. In addition, coyote diet selection and alternate prey resource availability in locations with otter‐mediated carrion subsidies would certainly influence these ecological responses, so future research efforts should attempt to estimate the contribution of otter‐provisioned carrion to coyote diets. Future efforts to quantify the role of river otter‐provisioned carrion on terrestrial food webs should examine the abundance, distribution, nutrient and caloric value, contaminant levels, and predictability of otter‐provisioned carrion subsidies in addition to the hypothesized ecological consequences we have presented. Such inquiry can help us better understand the mechanisms through which carrion provisioning by river otters elicits cascading impacts within and across ecosystems and broaden our understanding of the diverse ecological consequences of river otter recolonization.

Our results suggest that ongoing river otter population recoveries and range expansions will continue to generate novel species interactions, including with terrestrial kleptoparasites, which may produce subsequent shifts in ecosystem structure, function, and connectivity.

## AUTHOR CONTRIBUTIONS

**Francis D. Gerraty:** Conceptualization (equal); data curation (lead); investigation (lead); project administration (lead); visualization (lead); writing – original draft (lead); writing – review and editing (equal). **Terence Carroll:** Conceptualization (equal); data curation (supporting); investigation (supporting); writing – original draft (supporting); writing – review and editing (lead). **Skyler Williams:** Data curation (equal); investigation (supporting); project administration (supporting); visualization (supporting); writing – original draft (supporting); writing – review and editing (supporting). **Megan Isadore:** Conceptualization (equal); data curation (supporting); investigation (supporting); writing – original draft (supporting); writing – review and editing (equal).

## CONFLICT OF INTEREST STATEMENT

All authors declare that they have no conflicts of interests to disclose.

## Data Availability

All data supporting the findings of this study are presented within the manuscript.
